# Microdebrider Adenoidectomy Technique

**DOI:** 10.7759/cureus.113252

**Published:** 2026-07-23

**Authors:** Amer M Mansour, Abraham A Kassem, Chinelo Eruchalu, Tony Han, Michele M Carr

**Affiliations:** 1 Otolaryngology - Head and Neck Surgery, Jacobs School of Medicine and Biomedical Sciences, University at Buffalo, Buffalo, USA; 2 Otolaryngology, Icahn School of Medicine at Mount Sinai, New York, USA

**Keywords:** adenoidectomy, adenoid hypertrophy, coblation adenoidectomy, endoscopic adenoidectomy, microdebrider, nasopharynx, obstructive sleep apnea, otitis media, pediatric otolaryngology, surgical technique

## Abstract

Adenoidectomy is a common surgery among children in the United States. Adenoid hypertrophy is associated with several disorders that impact childhood development, such as sleep disordered breathing, otologic disease, and gastroesophageal reflux disease (GERD). The gold standard treatment for these disorders has been surgical excision of the adenoid tissue. Several techniques have been employed in the past, including sharp dissection with curettes, monopolar suction cautery, coblation, and microdebrider excision. Each of these techniques has advantages and disadvantages with respect to operative time, intraoperative blood loss, and postoperative complications, the most important of which are postoperative bleeding, adenoid regrowth, and velopalatal insufficiency. Here, we describe the use of a microdebrider for excision of the adenoid and explore the advantages and disadvantages of this method. The microdebrider technique is effective and safe, particularly for ENT surgeons who have significant experience using the microdebrider. This technique allows for precise tissue excision without thermal injury to surrounding structures. It also allows for full visualization of the posterior nasopharynx due to the microdebrider's built-in suction mechanism, which keeps the field clear of any tissue debris or blood that may accumulate during the excision. This experience mirrors much of the literature on this subject, which demonstrates the similar effectiveness of the microdebrider technique when compared with others and has identified a decreased risk of postoperative bleeding and adenoid regrowth.

## Introduction

Adenoidectomy remains one of the most commonly performed procedures in pediatric otolaryngology and is frequently indicated for conditions including obstructive sleep apnea (OSA), chronic otitis media with effusion (OME), nasal obstruction, and chronic rhinosinusitis [1-3]. Over time, several surgical techniques have been developed for adenoid removal, including curettage, electrocautery, coblation, suction cautery, and powered microdebrider-assisted approaches [2]. Among these techniques, microdebrider adenoidectomy offers enhanced visualization and precise tissue removal. This article reviews the anatomy and clinical significance of the adenoid and provides a detailed description of the microdebrider adenoidectomy technique.

Adenoid Anatomy and Function

The adenoid is a patch of lymphatic tissue covered in respiratory epithelium located in the nasopharynx. Along with the palatine and lingual tonsils, the adenoid constitutes the Waldeyer ring, a secondary lymphoid structure that defends the pharynx against upper airway invasion by many pathogens [4]. The tissues of this Waldeyer ring play a role in immunity through the development of B- and T-lymphocytes. The adenoid primarily acts to activate B-cells, and recent research has suggested that the adenoid is a site of T-cell production [4]. The specific placement of the adenoid in the upper airway allows it to sample pathogens that are introduced to the body via ventilation, allowing for improved surveillance and recognition of infectious microbes by immune cells [4].

Anatomically, the adenoid is situated midline on the superior, posterior wall of the nasopharynx. Bounded by the tori tubarii and fossa of Rosenmuller laterally, the adenoid is in close proximity to several important structures of the upper airway [4]. The Eustachian tubes, soft palate, choanae, and the uvula are all anatomically situated near the adenoid and therefore may be affected by this tissue. This anatomic location, while advantageous for immune surveillance, is the main factor underlying a multitude of aerodigestive disorders that are a consequence of adenoid hypertrophy.

Adenoid Hypertrophy Etiology, Epidemiology, and Associated Disorders

As children reach ages between five and seven, the adenoid rapidly enlarges, reaching its peak size at this age [5]. It is estimated that approximately 35% of children develop some level of adenoid hypertrophy [6]. As children age into adolescence, the immune system matures, making lymphoid tissues in the nasopharynx redundant. The adenoid, therefore, decreases in size, reducing this prominent lymphoid tissue in the nasopharynx and decreasing the prevalence of adenoid hypertrophy as patients age.

Adenoid hypertrophy has been associated with several infectious and non-infectious processes, which likely contribute to its development. These infections may be viral in nature and include common upper respiratory viruses, such as cytomegalovirus (CMV), Epstein-Barr virus (EBV), rhinovirus, herpes simplex virus (HSV), and parainfluenza virus [7]. Similarly, bacterial respiratory infections caused by pathogens such as *Streptococcus *species, *Haemophilus influenzae*, and *Fusobacterium *species have also been implicated in adenoid hypertrophy [8]. With respect to non-infectious etiologies, environmental factors seem to be the most common factors associated with adenoid hypertrophy. These factors include cigarette smoke, air pollutants, and allergens, all of which contribute to upper airway inflammation [9]. Additionally, gastroesophageal reflux disease (GERD) has been linked to the development of adenoid hypertrophy, as refluxed stomach acid can irritate the upper airway, leading to inflammation and edema [10].

Given the relatively small upper airway diameter in children, even mild adenoid hypertrophy may lead to several aerodigestive disorders. The most common sequelae result from obstruction of the nasal airway or Eustachian tubes by an enlarged adenoid [11]. Obstruction of the upper airway may cause difficulty breathing through the nose and sleep-disordered breathing, such as OSA [11,12]. Several studies have demonstrated significant complications associated with OSA in pediatric populations. The most clinically relevant of these, due to their prevalence or severity, are pulmonary hypertension, cognitive impairment, and failure to thrive [12-14].

In addition to upper airway complications, adenoid hypertrophy may contribute to otologic disease through obstruction of the Eustachian tubes. Mechanical obstruction of the Eustachian tube may lead to OME or recurrent acute otitis media due to decreased drainage of fluid from the middle ear space. This may lead to difficulty hearing, speaking, and learning [15]. Further potential downstream sequelae of OME are tympanic membrane perforation, cholesteatoma, and conductive hearing loss [16].

Beyond upper airway and otologic sequelae, adenoid hypertrophy has also been associated with gastrointestinal disorders, including GERD, esophagitis, and dysphagia [17-21]. It is theorized that the airway obstruction caused by adenoid hypertrophy leads to increased negative thoracic pressure as the lungs attempt to overcome the obstruction. At the same time, abdominal pressure increases, thereby increasing the risk of reflux into the esophagus [17,18,21]. Additional studies have also found that GERD symptoms improve following adenoidectomy in patients with adenoid hypertrophy, further supporting the theory that adenoid hypertrophy can increase the risk of GERD [22-24].

Additionally, the increased tissue volume that adenoid hypertrophy introduces into the aerodigestive tract has been associated with dysphagia and sialorrhea in young patients [25]. An enlarged adenoid may reach the oropharynx, physically blocking the passage of food through the upper aerodigestive tract [25]. Similar to findings regarding GERD following adenoidectomy, studies have demonstrated significant improvement in dysphagia symptoms after adenoidectomy [26,27].

Adenoidectomy History and Indications

Adenoidectomy, the surgical excision of the adenoid, was first pioneered in the 19th century by Hans Wilhelm Meyer, and it continues to be one of the most common pediatric surgical procedures [1]. A 2023 review of tonsillectomy and adenoidectomy rates across the United States reflects this trend, with an estimated 500,000 procedures performed annually in children less than 15 years of age [2]. The primary indications for an adenoidectomy include chronic OME, OSA, nasal obstruction in the presence of a large adenoid, chronic adenoiditis, and recurrent rhinosinusitis. Coupled with it being a simple, cost-effective procedure, adenoidectomy has become a first-line surgical treatment for children up to six years of age with chronic rhinosinusitis [3].

Adenoidectomy Complications

The risks following an adenoidectomy are most commonly pain post-surgery, dehydration, fever, regrowth of the adenoid, hemorrhage, and rarely velopharyngeal insufficiency (VPI) [28-30]. Pain after surgery typically manifests as throat, neck, jaw, or ear pain, with dehydration occurring secondary.

Adenoid regrowth post-adenoidectomy can be a source of ongoing symptoms and need for further therapy. Although the regrowth of adenoid (significant enough to cause symptoms) is between 1% and 8%, it is reported to be more common in children younger than five who were treated postoperatively with numerous antibiotics [31-33].

Hemorrhage is divided into primary (within the first 24 hours following surgery) and secondary (over 24 hours post-surgery). Primary hemorrhage risk is higher than secondary, with a 0.5%-0.8% risk within 24 hours following surgery [34].

VPI is a rare post-surgical complication manifested by hypernasal speech and nasal regurgitation caused by dysfunction of the velopharyngeal sphincter, which separates the nasal and oral cavities. It has a reported incidence of one in 1,500 patients and is more common in patients with hypotonia or submucosal cleft palate [29,30].

Summary of Adenoidectomy Techniques 

Various instruments can be used to excise the adenoid. Methods of adenoidectomy include the use of a curette, monopolar suction cautery, coblation device, powered microdebrider, or laser. The curette technique remains widely used due to its accessibility and shorter surgical times. In a prospective observational study by Ferreira et al. in 2018, 33 children were divided into three groups based on the technique used: Group A (curette, 18 children), Group B (microdebrider, 8 children), and Group C (radiofrequency ablation, 4 children) [35]. While Group A had the shortest surgical time (9.6 minutes vs. 13.6 minutes for Group B), there were significant limitations. Despite its efficiency, the curette technique lacks direct visualization, increasing the likelihood of residual adenoid tissue following surgery. Ark et al. in 2010 reported that 79 of 99 patients who underwent curette adenoidectomy had residual tissue, with 81% of these near the choanae, which led to postoperative complications. such as bleeding and persistent nasal obstruction [36].

Suction electrocautery, also known as suction diathermy, has gained popularity due to its lower bleeding risk and its ability to provide more precise tissue removal. This technique uses heat generated by electric currents to remove adenoid tissue while simultaneously sealing blood vessels. However, it is associated with some issues, such as postoperative neck pain, Eustachian tube dysfunction, the risk of burns to oral tissues, and the potential for adenoid regrowth. A 2007 retrospective study by Skilbeck et al. reviewed 1,387 patients who underwent suction diathermy adenoidectomy over 10 years. Postoperatively, 24 patients still experienced symptoms, with suspected adenoid tissue regrowth [37]. A meta-analysis by Reed et al. in 2009 assessed the effectiveness of electrocautery adenoidectomy (ECA) in pediatric patients. The study found that ECA resulted in shorter surgery times compared to the traditional curettage technique (10.0 minutes vs. 18.4 minutes) [38]. The analysis included data from nine studies with a mean sample size of 276 patients. However, adenoid regrowth was reported in seven studies, and postoperative complications, such as bleeding and infections, were observed in eight studies [39].

Coblation is another technique that uses controlled low temperatures to remove tissue with minimal damage to surrounding tissues. In a prospective study by Bidaye et al., 60 children participated in a study comparing the cold curette technique to the coblation technique [39]. They found that coblation had a longer surgical time due to complex setup requirements and the need for nasal cavity decongestion. Additionally, no ablated tissue was available for histological examination, unlike the microdebrider or curette techniques [39].

## Technical report

The microdebrider technique is a precise and controlled approach to adenoidectomy, using a rotating blade under endoscopic guidance or mirror visualization. It can be performed either transorally or transnasally. A unique feature of the technique is the integrated pedal system, which allows the surgeon to control the speed and direction of the blade during the procedure. Additionally, the built-in suction mechanism removes debris, maintaining a clear surgical field and improving visibility [40].

This technique, which will be described in more detail, demonstrates several advantages over the suction cautery, coblation, electrocautery, and curette techniques. Unlike suction cautery, the microdebrider technique is associated with a lower risk of thermal damage to surrounding tissues, allowing for more precise tissue removal [35,41]. Similarly, the microdebrider's controlled, directly visualized approach reduces the risk of bleeding and damage to surrounding nasopharyngeal structures. This, therefore, ensures more complete resection and fewer complications. Additionally, the microdebrider technique is associated with a decreased risk of airway fire due to its limited use of electrocautery. Finally, the cost of the equipment also made coblation less accessible in some healthcare settings when compared with the microdebrider technique [35,39].

Microdebrider Technique Steps

**Figure 1:**
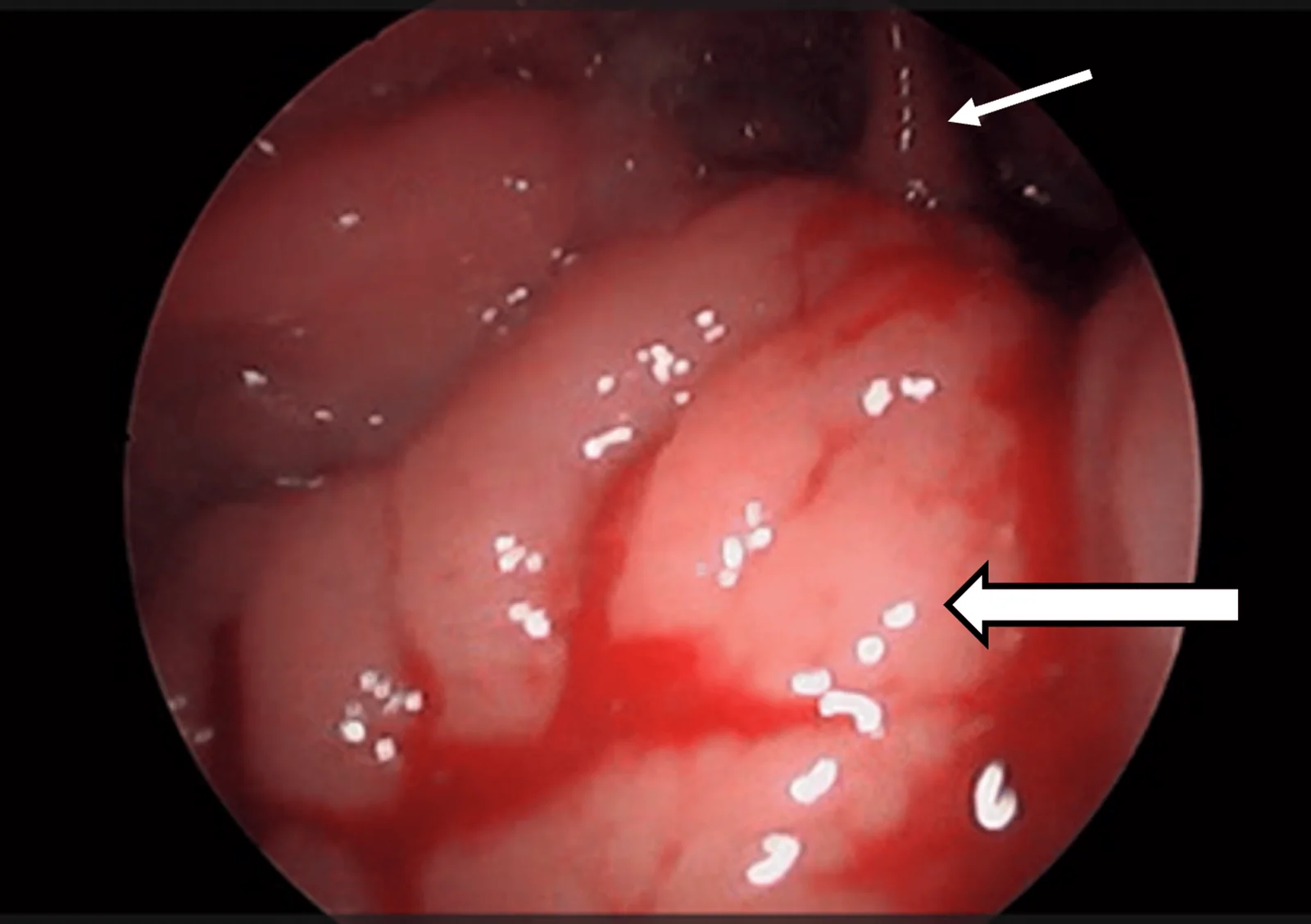
View of the adenoid via mirror placed transorally. Large arrow: adenoid; small arrow: posterior vomer

**Figure 2:**
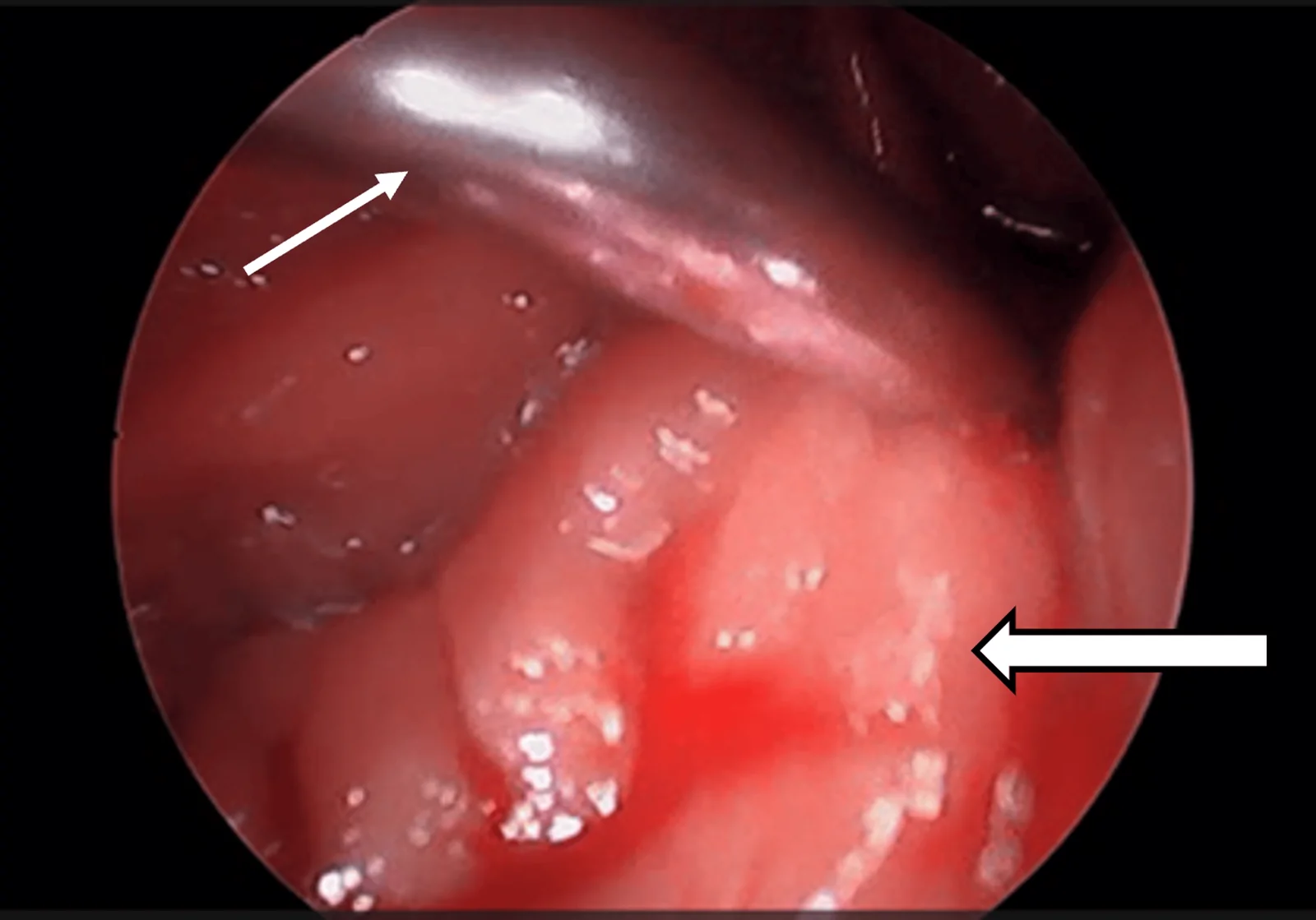
Starting the dissection with the microdebrider. The adenoid blade is placed anterior to the adenoid, near the choana, cutting away from the nose. Large arrow: adenoid; small arrow: microdebrider

The patient is laid supine on the operating room table; anesthesia is induced, and a Ring-Adair-Elwyn (RAE) endotracheal tube is placed. A shoulder roll is placed for children. The Crowe-Davis or McIvor mouth prop is placed, and suspension is applied. The oral commissures are coated with a water-based lubricant. A rubber, silicone, or plastic catheter is placed into the nasal cavity, passed down to the pharynx, brought out through the mouth, and clamped over a rolled 4x4 gauze sponge to suspend the palate and improve visualization of the nasopharynx. The microdebrider adenoidectomy blade (RADenoid small blade, 11x4 cm, 40 ° blade tip, ref 1884008; Medtronic Xomed, Inc., Jacksonville, FL) is fit onto the handpiece and tested. Rotations are set to 2,000-2,500 per minute. A dental mirror is used to visualize the adenoid tissue in the nasopharynx (Figure 1). The microdebrider is used to remove the tissue, taking care to avoid abrading the peri-Eustachian tube tissue, the torus tubarius. Placing the cutting edge of the microdebrider into the choana and cutting toward Passavant's ridge allows for complete excision of tissue filling the choanae (Figure 2). The most proximal part of the adenoid, closest to Passavant's ridge, is left intact. The cut edge is beveled so that the entire cut surface can be seen with the dental mirror.

**Figure 3:**
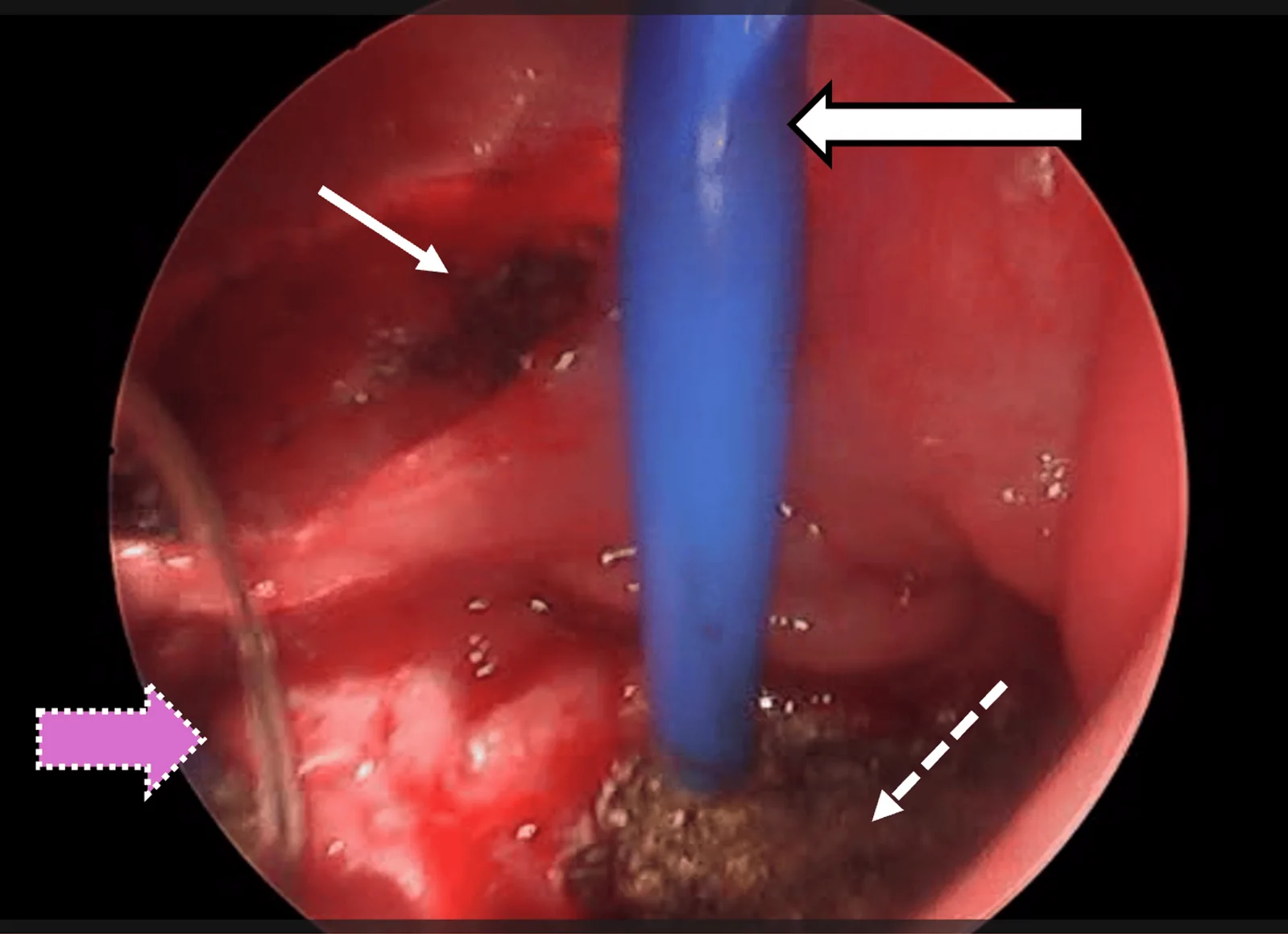
Completing the microdebrider adenoidectomy. Bleeding was controlled with the suction cautery. Large arrow: suction cautery; small arrow: tonsil fossa; small dashed arrow: cauterized adenoid beds; large dashed arrow: dental mirror

The nasopharynx is then packed with gauze tonsil balls lightly covered with oxymetazoline nasal spray. These are left in place for at least a minute and then removed. The area is irrigated with saline water and suctioned clear. Suction cautery set at between 30 and 40 W is used for hemostasis (Figure 3). The catheter and mouth prop are removed, and the patient is returned to the anesthesia team for awakening. The complete surgical technique is demonstrated in Video 1.

**Video 1:** Microdebrider adenoidectomy technique. This video shows the complete transoral microdebrider adenoidectomy procedure.

## Discussion

The current literature on the use of the microdebrider for adenoidectomy supports the effectiveness of this technique. In 2010, Somani et al. reported high surgeon satisfaction in their study of adolescents undergoing adenoidectomy with the microdebrider, emphasizing the completeness of tissue removal [42]. Similarly, Wadhera et al. in 2022 found that all 20 patients undergoing microdebrider adenoidectomy had complete tissue removal with no complications, such as nasal obstruction or postoperative bleeding, demonstrating its advantages over traditional adenoidectomy methods [41]. Furthermore, a 2023 study by Bakshi et al. demonstrated the efficacy of the microdebrider technique with respect to complete adenoid removal. The study reported significant improvement in snoring, nasal obstruction, and breathing after microdebrider adenoidectomy [43]. Similarly, a retrospective analysis of more than 450 patients demonstrated that the microdebrider technique is associated with a lower risk of certain postoperative adverse effects, including neck pain, halitosis, and fever, when compared with coblation [44].

In contrast to these studies, there has been some reported data suggesting that the microdebrider technique results in greater operative blood loss and longer surgical times when compared with traditional curettage. A 2023 systematic review and meta-analysis presented data showing increased intraoperative blood loss and operative time for the microdebrider technique when compared with the suction cautery and cutterage techniques [2]. On the other hand, this study demonstrated a decreased rate of residual adenoid tissue in patients who underwent microdebrider adenoidectomy compared to curettage adenoidectomy [2]. Consistent with these results, a comparative study by Modi et al. showed that the microdebrider technique was associated with decreased rates of residual adenoid tissue but greater surgical time and intraoperative bleeding when compared with conventional curettage adenoidectomy [45].

Further studies demonstrate limited differences between the techniques discussed, and these data suggest that the choice of technique should be determined by the surgeon's familiarity and experience. Singh et al. presented data comparing the coblation technique with the microdebrider technique, showing no statistically significant difference between the two with respect to postoperative complications or completeness of adenoid tissue removal [46].

In summary, the data suggest that, although the microdebrider technique may be marginally slower and may be associated with slightly greater operative blood loss, it is also associated with decreased risk of adenoid regrowth and postoperative complications. These considerations suggest that the microdebrider technique is a useful, effective, and safe option for adenoidectomy, particularly for surgeons who are highly experienced and trained in this technique.

## Conclusions

The adenoid, lymphatic tissue in the nasopharynx, plays a critical role in defending against upper airway infections but can also be associated with chronic otitis media and obstructive sleep apnea. The microdebrider technique provides a controlled and precise approach to adenoidectomy, utilizing a rotating blade under endoscopic guidance or direct visualization for effective and precise tissue removal. Studies have demonstrated its efficacy in achieving complete adenoid resection with minimal damage to surrounding structures, reducing the incidence of postoperative complications such as bleeding and nasal obstruction.

The microdebrider technique is a valuable addition to the adenoidectomy armamentarium. Overall, it provides benefits in terms of precision, safety, and patient outcomes when employed by experienced surgeons, making it an essential option for managing adenoid-related conditions in clinical practice.
